# Is bullying in adolescence associated with the development of depressive symptoms in adulthood?: A longitudinal cohort study

**DOI:** 10.1186/s40359-020-00491-5

**Published:** 2020-11-19

**Authors:** Trine Nøhr Winding, Lisbeth Astrid Skouenborg, Vibeke Lie Mortensen, Johan Hviid Andersen

**Affiliations:** Department of Occupational Medicine–University Research Clinic, Danish Ramazzini Centre, Regional Hospital West Jutland, Gl. Landevej 61, 7400 Herning, Denmark

**Keywords:** Bullying, Depressive symptoms, Adolescence, Prospective study

## Abstract

**Background:**

Being bullied in adolescence is linked to mental health problems like anxiety, depressive- and somatic symptoms and can have negative consequences on both an individual and a societal level. However, evidence regarding the long-term mental health consequences of bullying in adolescence is limited. The aim of this study was to examine whether being bullied at age 15 or 18 was associated with experiencing depressive symptoms at age 28, and to examine whether being bullied at both ages 15 and 18 increased the risk of experiencing depressive symptoms at age 28.

**Methods:**

A prospective cohort study, which applied data from the West Jutland Cohort Study, was conducted. Bullying and depressive symptoms were measured on the basis of self-reported data from surveys in 2004, 2007 and 2017. Depressive symptoms were measured with the Center for Epidemiological Studies Depression Scale. A total of 1790 participants were included in the study, and analyzed by multiple logistic regressions.

**Results:**

The results showed associations between being bullied at age 15 or 18 and the reporting of depressive symptoms at age 28 when adjusted for potential confounders. An exposure–response relationship was seen in those who were bullied at both ages 15 and 18. This group had the highest risk of developing depressive symptoms at age 28.

**Conclusions:**

Being bullied in adolescence was associated with developing depressive symptoms in adulthood and there was an exposure–response relationship between being bullied over time and the later reporting of depressive symptoms. The results highlight the need to provide more detailed information to schools and local communities about the negative consequences of bullying. Such increased awareness may help reduce the risk of young people developing depressive symptoms later in life.

## Introduction

Depression is one of the most common mental illnesses and the number of people with mental health problems is increasing worldwide. Over the past 20 years, the number of mental health problems among young people in particular has risen considerably and account for most of the burden of illness for this age group [1].

Characteristics of depression are sadness, a poor state of mind, feelings of guilt, decreased self-esteem and difficulty sleeping [1]. The causes of depression are a complex interplay of social, psychological and biological factors. In addition to the immediate individual consequences of mental health problems, there is also a growing recognition that these problems have a large compromising influence on the individual's ability to cope educationally, professionally and financially and thus reduce the individual's ability to contribute to society [2, 3]. The prevention and treatment of mental health problems is therefore of great importance and interest on both an individual and a societal level.

The psychological well-being of children and young people in Denmark is declining. A recent report shows that levels of psychological satisfaction among young people, especially teenage girls, have decreased and that more children and young people are reporting conflicts with peers [4]. Today every fifth child in Denmark is exposed to bullying [5]. Bullying is a social phenomenon and in Denmark the proportion of people being subjected to bulling is highest among 11 year olds (11%) and falls to 4% among 15 year olds [6]. Although the incidence of bullying generally decreases from childhood to adolescence, bullying may be more targeted and persistent in adolescence [7]. Socializing with peers becomes more important during adolescence, which is a particularly sensitive life period, characterized by many and rapid social, emotional and physiological changes [8]. Bullying during this period may therefore have a particularly adverse effect on mental health extending into adult life [9]. Bullying is associated with several adverse outcomes later in life, such as reduced self-esteem, reduced desire to engage in social relations, and health problems such as anxiety, depression and psychosomatic disorders [10–14]. Several cross-sectional studies indicate that there is an association between bullying and mental health problems among children and adolescents [12–17], and longitudinal studies have linked bullying at school to depression and depressive symptoms in adolescence [7, 17–19]. However, there is still a relatively limited amount of research that examines the long-term consequences of bullying in childhood and adolescents and most studies have limited follow-up periods that do not extend into adulthood [7, 18, 19]. A few longitudinal studies identify a link between childhood bullying and mental health problems in adult life [10, 20]. A British longitudinal study, spanning five decades, found that children who were frequently bullied had a higher risk of anxiety disorders, depression and suicide at age 23 and 50 relative to peers who had not been bullied [10]. Sigurdson et al. [20] found that bullying among Norwegian adolescents at age 14/15 was associated with mental health problems at age 27 and that those who had been exposed to bullying had a significantly greater need for psychiatric help as adults compared with those who had not been bullied. In contrast, in a twin study, Singham et al. [21] showed that the negative effect of bullying on mental health diminished over time among children and adolescents, which Östberg et al. [22] also found, but only for males. The literature lacks information about the consequences of more persistent bullying during childhood and adolescence for mental health later in life. At the same time those being exposed to bullying report having fewer close friends and a poorer relation to their parents compared to those not exposed to bullying [16, 21]. A study by Bjereld et al. [13] found that among children aged 4–16 from Nordic countries, those with many close friends had higher odds to be mentally healthy than children with fewer close friends.

This altogether indicates a divergence in the results of the existing literature.

The sparse national and international evidence regarding the long-term mental health consequences of bullying in adolescence combined with the divergence in the results of the existing literature makes it highly relevant to examine the association between adolescent bullying and the development of depressive symptoms in early adult life.

## Methods

### Aim

The primary aim of the present study was to investigate whether exposure to bullying at ages 15 or 18 was associated with the development of depressive symptoms at age 28.

The secondary aim was to investigate whether exposure to bullying at both ages 15 and 18 increased the risk of developing depressive symptoms at age 28.

### Design and population

This is a longitudinal study using questionnaire data gathered as part of the ongoing West Jutland Cohort Study (VestLiv), which aims to investigate aspects of inequality in health and social differences in welfare from a lifelong perspective [23, 24]. Individuals born in 1989 who were living in the county of Ringkjoebing (West Denmark) in April 2004 (age 15) were invited to participate (N = 3681). Contact information for this complete regional cohort of young people was retrieved from the Central Office of Civil Registration and from public schools in the county of Ringkjoebing. Of the original source population, 3054 (83%) filled out the initial questionnaire at age 15 in 2004.

Information from two follow-up surveys were used at ages 18 and 28 with response rates of 65% (n = 2400) and 57% (n = 2102) respectively. Register information about the respondents was derived from national registers in Statistics Denmark using the personal identification number from the Central Office of Civil Registration (CPR number), which is given to every inhabitant in Denmark at birth (or upon entry for immigrants) [25]. To obtain information about parental educational level, gender and family type (split home), the respondents were also linked to their parents or guardians using the CPR number [25].

**Fig. 1:**
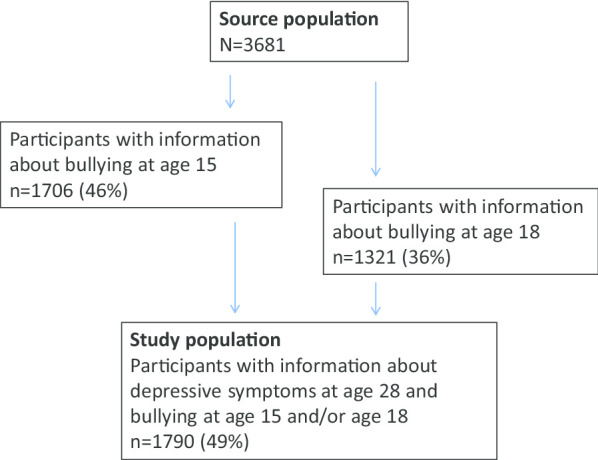
Inclusion into the study population

The study population consists of participants who provided information about depressive symptoms at age 28 and bullying at age 15 and/or age 18 (n = 1790). The response rates of the participants in years 2004, 2007 and 2017 are presented in Fig. 1.

### Outcome

The outcome of the study was depressive symptoms, which was based on the participants' self-reporting at age 28. We included four items from The Center for Epidemiological Studies Depression Scale (CES-D). This scale is an abbreviated validated version of the original scale [26, 27] and is designed to measure the current level of depressive symptoms in a general population, with emphasis on the affective component “depressed state”. The CES-D scale was developed for use in epidemiological studies [27] and has been translated into several languages and validated for both young people and adults [26, 28]. Participants were asked the following 4 questions to assess their level of depressive symptoms: During the past week, how often have you had the following feelings?": (1) "I was happy", (2) "People were unfriendly", (3) "I felt sad", (4) "I could not get going" with the following response options: (1) "Not at all", (2) "A little", (3) "Some", (4) "A lot". The responses were subsequently awarded scores of 0–3 and generated into a sum-score ranging from 0–12, with high values corresponding to having more depressive symptoms. The scales were then dichotomized at 3 points and above into few depressive symptoms and more depressive symptoms, as suggested by Fendrich et al. [26], who found this cut-point relevant in relation to the prediction of major depressive disorders in the general population.

### Exposures

Questionnaire information about bullying was obtained by the participants' self-reporting at age 15 and 18. At age 15, the participants were asked: "How much have you been bullied at school during the last six months?". At age 18, the participants were asked about bullying in a slightly different way: "How much have you been bullied in an unpleasant way at school during the last six months?". At both ages, the response options were: (1) "Never", (2) “Once or twice”, (3) “A few times”, (4) “Once a week”, (5) "Several times a week". These options were combined into the following three categories: "Not bullied" if they had answered (1), "Bullied" if they had answered (2) or (3) and "Often bullied" if they had answered (4) or (5), as suggested by Andersen et al. [29].

### Potential confounders

Information about potential confounders was obtained from the participants' questionnaire responses at age 15 and 18 and from register information.

Information about the highest level of education in the household in 2003 was obtained from the educational registers [30] and divided into the following 4 categories: (1) ≤ 10 years (primary school), (2) 10–13 years (secondary school), (3) 13–15 years (short/middle tertiary education), and (4) > 15 years of school (long tertiary education). If the participants’ parents were divorced, information was taken from the household at which the participants had their postal address.

Information about close friends was measured as a question, at age 15, about whether the participants had a friend in whom they could confide (yes vs. no).

Information about family functioning was based on the participants' responses to questions regarding the general functioning of the family at age 15. The General Functioning Scale consists of 12 items that assess the overall health or pathology of the family and is one of seven scales from the McMaster Family Assessment Device (FAD) [31]. In this study, the variable was dichotomized at a cut-off of ≥ 2, corresponding to the 75%-percentile, with high scores indicating a problematic family function [29, 31].

Information about split home and gender was collected from registers [25]. The variable split home was dichotomized into whether the participant lived with one or both parents.

### Statistical methods

The characteristics of the study population were presented by gender, and the distributions of the categorical variables were presented by number and proportion.

The distributions of being bullied at age 15 and 18 in relation to depressive symptoms at age 28 were presented by number (n) and proportion (%). Because only a limited number of participants reported that they were "often bullied" at age 18, the categories "Bullied" and "Often bullied" were collapsed into one category called "Bullied" in regard to all analyses using information about bullying at age 18.

The associations between being bullied at age 15 or 18 and the development of depressive symptoms at age 28 were analyzed using multiple logistic regression. Firstly, crude estimates between each exposure variable and outcome were performed. Secondly, estimates adjusted for parental educational level, close friends, family functioning, gender and split home were calculated. Thus, crude and adjusted odds ratios (ORs) were estimated with 95% confidence intervals (95% CI). As supplementary analyses, the associations between being bullied at age 15 or age 18 and depressive symptoms at age 28 was adjusted for depressive symptoms at age 15.

Finally, the association between being bullied both at age 15 and age 18 and developing depressive symptoms at age 28 was investigated by constructing the following three categories:

- "Not bullied", if not bullied at age 15 or 18
- "Bullied at one age point", if Bullied or Often bullied at either age 15 or 18,
- "Bullied at two age points", if Bullied or Often bullied at both age 15 and 18

We furthermore carried out a sensitivity analysis using a multiple imputation chained model with 100 imputations. Seven chains, either logit or ologit dependent on the categorization of the variables, were constructed in order to impute missing data on the following variables: depressive symptoms at age 28, bullying at age 15 or 18, family functioning and close friends at age 15, split home and household educational level in year 2003. Information on gender was complete. Besides the above mentioned variables some additional were included: (1) depressive symptoms at age 15 and 18 were included when imputing depressive symptoms at age 28, (2) being bullied at age 28 was included when imputing bullying at age 15 and 18, respectively, The final estimates were found as the average of the *m* sets of estimates and the standard errors by applying a simple formula called Rubin’s rule (results not shown) [32]. All statistical analyses were carried out in STATA statistical package (V.15.0; State, College Station, TX).

### Ethical considerations

This study is in accordance with the 1975 Declaration of Helsinki [33]. The study is approved by the Danish Data Protection Agency. According to Danish law at the time point of data collection, questionnaire and register-based studies did not need written informed consent nor approval by ethical or scientific committees [34].

## Results

Characteristics of the study population are presented in Table 1.

**Table 1:** Characteristics of the study population: n = 1790, by gender

	Total n = 1790	Males n = 746 (42%)	Females n = 1044 (58%)
	n (%)	n (%)	n (%)
Depressive symptoms, age 28			
Few	1033 (58)	447 (60)	586 (56)
More	757 (42)	299 (40)	458 (44)
Bulling, age 15			
Not bullied	1277 (75)	523 (74)	754 (75)
Bullied	388 (23)	168 (24)	220 (22)
Often bullied	41 (2)	12 (2)	29 (3)
Bulling, age 18			
Not bullied	1173 (89)	448 (87)	725 (90)
Bullied	148 (11)	66 (13)	82 (10)
Close friends, age 15			
Yes	1519 (89)	594 (85)	925 (92)
No	182 (11)	105 (15)	77 (8)
Family functioning, age 15			
< 2	1146 (70)	475 (70)	671 (70)
≥ 2	494 (30)	200 (30)	294 (31)
Split home year 2003			
Living with both parents	1399 (78)	612 (82)	787 (76)
Living with one parent	390 (22)	134 (18)	256 (25)
Household educational level year 2003			
< 10 years	198 (11)	78 (11)	120 (12)
10–12 years	907 (51)	353 (48)	554 (54)
12–15 years	542 (31)	253 (34)	289 (28)
> 15 years	124 (7)	55 (7)	69 (7)

Table 1 shows that more females than males participated in the study. Slightly more females than males reported more depressive symptoms. There was a decrease in the prevalence of bullying from age 15 to age 18 in both females and males. 26% of males were bullied ("bullied" or "often bullied") at age 15 and this level dropped to 13% at age 18. 25% of females were bullied ("bullied" or "often bullied") at age 15 and this level dropped to 10% at age 18. The prevalence of males who did not have any close friends at age 15 was higher compared with the females and the prevalence of males who were living with both parents was higher compared with the females.

Table 2 shows that more participants who had been "bullied" or "often bullied" at ages 15 or 18, reported more depressive symptoms at age 28 than those who had not been bullied.

**Table 2:** Distribution of being bullied at age 15 and 18 in relations to depressive symptoms at age 28 presented by number n and proportion (%)

	Depressive symptoms		
	Total	Few	More
	n	n	n
Bullied, age 15, n = 1706			
Not bullied	1277	780 (61)	497 (39)
Bullied	388	196 (51)	192 (49)
Often bullied	41	16 (39)	25 (61)
Bullied, age 18, n = 1321			
Not bullied	1173	721 (61)	452 (39)
Bullied	148	65 (44)	83 (56)

Table 3 shows OR in the range of 1.6 to 2.1 between being bullied at age 15 or age 18 and the reporting of depressive symptoms at age 28, when adjusted for potential confounders.

**Tabel 3:** The association between being bullied at age 15 or 18 and depressive symptoms at age 28, N = 1790

	Crude estimates		Adjusted estimates^a^	
	OR	95% CI	OR	95% CI
Bullied, age 15				
Not bullied	Ref		Ref	
Bullied	1.5	1.2; 1.9	1.6	1.2; 2.0
Often bullied	2.5	1.3; 4.6	1.8	0.9; 3.6
Bullied, age 18				
Not bullied	Ref		Ref	
Bullied	2.0	1.4; 2.9	2.1	1.4; 3.0
Gender				
Male	Ref			
Female	1.2	1.0; 1.4		
Close friends, age 15				
Yes	Ref			
No	0.96	0.7; 1.3		
Family functioning, age 15				
< 2	Ref			
≥ 2	1.6	1.3; 2.0		
Split home year 2003				
Living with both parents	Ref			
Living with one parent	1.3	1.0; 1.6		
Parental educational level				
< 10 years	1.1	0.7; 1.7		
10–12 years	0.6	0.4; 0.9		
12–15 years	0.7	0.5; 1.1		
> 15 years	Ref			

^a^Adjusted for gender, close friends, family functioning, split home and parental educational level

Being bullied at age 15 increased the risk of reporting depressive symptoms by 1.6 and reporting being often bullied at age 15 increased the risk by 1.8, though statistical insignificant, when adjusted for potential confounders. Being bullied or often bullied (one category) at age 18 increased the risk of reporting depressive symptoms at age 28 by around 2 compared with those who had not experienced bullying.

Adjusting the association between being bullied at age 15 and depressive symptoms at age 28 for depressive symptoms at age 15 changed the adjusted OR to 1.6 (1.2; 2.0) in "bullied" and to 1.4 (1.1; 1.8) in "often bullied", and the adjusted OR between "bullied" age 18 and depressive symptoms age 28 changed to 2.0 (1.4; 3.0).

Table 4 shows a clear exposure–response relationship between the extents of bulling over time.

**Table 4:** The association between being bullied at one age point (age 15 or 18) or two age points (age 15 and 18) and depressive symptoms at age 28, N = 1790

	Crude estimates		Adjusted estimates^a^	
	OR	95%-CI	OR	95%-CI
Not bullied	Ref		Ref	
Bullied at one age point	1.8	1.4; 2.3	1.8	1.3; 2.3
Bullied at two age points	2.5	1.5; 4.4	2.7	1.5; 4.8

^a^Adjusted for gender, close friends, family functioning, split home and parental educational level

Those who were bullied at one age point (age 15 or 18) had a 1.8-fold increased risk of reporting depressive symptoms at age 28, whereas those who were bullied at both age points (15 and 18) had a 2.7-fold increased risk of reporting depressive symptoms at age 28 compared with those who had not experienced bullying at either of the two age points. Both adjusted ORs decreased by 0.2 when also adjusting for depressive symptoms at age 15.

## Discussion

In this study we found that those who were bullied at ages 15 or 18 had an increased risk of developing depressive symptoms at age 28 compared with those who were not bullied. An association between being bullied and developing depressive symptoms already began to emerge among those who reported being bullied “once or twice” or “a few times” during the previous 6 months at age 15. However, the strongest association was seen among those who reported being bullied at both age 15 and 18.

Previous studies have documented an association between being bullied as a child and developing subsequent mental health problems [9, 16, 35, 36]. However, these studies vary greatly in their study design, follow-up periods, and definitions and categorizations of exposure and outcome. Moreover, studies investigating persistent bullying during childhood and adolescence are limited and only a handful of studies examine the long-term health consequences of bullying, which makes a direct comparison with the results of this study difficult. In the following discussion, the results of this study will therefore be compared with the longitudinal studies considered most relevant.

In line with our findings Takizawa el al [10] demonstrated that a high frequency of bullying had a negative impact on mental health. Likewise, Sourander et al. found that exposure to frequent bullying at age 8 was associated with severe psychiatric problems, including depression, as an adult [36]. However, the present study found that even infrequent bullying—“once or twice” or “a few times” during the previous 6 months—had a negative effect on the participants’ mental health.

Zwiezynska et al. [7] documented an exposure–response relationship between being bullied both at age 8 and 10 and symptoms of depression. These findings are in line with the findings of this study, which demonstrates an association between being exposed to bullying both in early and late adolescence and developing depressive symptoms in early adulthood.

Sigurdson et al. [20] showed an association between bullying and depressive symptoms, when adjusting for baseline depressive symptoms. The latter finding is consistent with the results of the present study, as adjustment for baseline depressive symptoms did not change the results significantly. By contrast, Zwiezynska et al. [7] found that depression at baseline had a significant effect on the development of depressive symptoms as an adult.

Previous studies have shown that more females than males experience depressive symptoms [7] and studies also reveal that both friendship and the type of friendship are important in relation to bullying and depressive symptoms. Skrzyipec et al. found that the likelihood of a person experiencing mental health problems decreased the more close friends he/she had [37]. This is supported by Bjereld et al. who found that bullied children who had more than 3 close friends were more likely to enjoy good mental health than bullied children with fewer close friends [13]. In this study, we made adjustments for background characteristics, gender, and social relations to family and peers, but these factors only explained a minor part of the association between being bullied during adolescence and reporting depressive symptoms as an adult.

### Strength and limitations

This study benefitted from a prospective design and a relatively large sample size. Information about bullying was collected from two separate age points, ages 15 and 18, which allowed us to examine the course of bullying throughout adolescence.

However, it is also important to note some limitations of the study. A main limitation was that both exposures and outcome were based on self-reporting, which increases the risk of common methods bias [38]. It is possible that participants who were bullied were also more likely to report depressive symptoms due to their mental state. This increases the risk of overestimating the associations between the exposures and the outcome and hence bias away from the null-hypothesis.

However, adjustments for depressive symptoms at age 15 did not change the estimates considerably, with a maximum change in OR of 0.4, why we believe that the risk of bias was minor.

Although the study showed significant associations between being bullied and experiencing depressive symptoms, is it important to be cautious of causal inference. Firstly, it could be argued that a 10-year gap between the latest exposure measurement of bullying and the measurement of depressive symptoms is too long. However, a certain amount of time between baseline and follow-up data is necessary as a main criterion of the definition of being bullied is the prolonged nature of the negative experience [39], and negative acts often develop over a long time span [40].

Another limitation of this study is missing information about bullying at age 18. Participating in surveys may be prone to selection bias if non-participation is associated with both exposures and outcomes. In this study, we found more participants were females, had higher educated parents and more often lived with both parents compared with the non-participants. However, non-participants and drop-outs in the same cohort were examined in a previous study and results showed that neither non-participants nor drop-outs influenced the size of the associations significantly [41]. We furthermore conducted a sensitivity analysis using a multiple imputation model (results not shown). These results showed none or small deviations of the adjusted estimates in both directions. The OR between bullying age 15 and depressive symptoms age 28 decreased in "bullied" from 1.6 to 1.5 (1.2; 1.9) and increased in "often bullied" from 1.8 to 2.2 (1.2; 4.2), whereas the adjusted estimate related to bullying at age 18 did not change. The adjusted estimate between being "bullied at one age point (age 15 or 18)" decreased from 1.8 to 1.7 (1.3;2.2), whereas the adjusted estimate related to "bullied at both age points (15 and 18)" decreased from 2.7 to 2.6 (1.5; 4.3). However, the assumptions of missing at random cannot be fulfilled with the imputation model, as the mechanism behind loss to follow-up in this study is unknown and may be related to unmeasured factors not included in the model. Furthermore, it is also worth noting that the measurement of bullying consisted of a single item, which allowed the individual participant to define the concept of bullying. An initial definition of bullying could have been beneficial, since the perception of what bullying is may be individual. Using such a definition could have increased the validity of the measuring tool. In relation to measuring exposure, it would have been beneficial to use a more in-depth questionnaire to determine the degree and nature of bullying but also to establish whether the participant was a victim or a perpetrator of bullying, since some studies show that both the bullied and the bully have a greater risk of mental health problems later in life [35, 36, 42].

In this study, there were few participants who were "often bullied" at age 18. This made it necessary to collapse the two bullied categories into one. This favored anonymity and increased the statistical strength, but it also made it more difficult to examine the exposure–response relationship between the amount of bullying at age 18 and depressive symptoms at age 28.

The questions used to measure depressive symptoms were derived from the CES-DC questionnaire [26]. We chose to dichotomize the scale in order to simplify the interpretation and increase the comprehensibility of the results. Although the proportion of young people with depressive symptoms seemed high, it does not differ much from findings in other populations [43, 44].

It is important to emphasize that this questionnaire measures symptoms and is not a well-established diagnosis of depression. The high proportion of young people presenting with depressive symptoms could reflect that early adulthood is a challenging period of life due to leaving home and starting working life or further education. We used a cut point of 3, but changing this cut point to 2 or 4 did not change the size of the estimates significantly.

The potential confounders were selected a priori based on a literature review. Additional potential confounders for the association between bullying and depressive symptoms could be ethnicity and personality type, which it would have been beneficial to adjust for in the current study.

The fact that the study is based on a complete regional cohort of young people and contains a considerable number of responders strengthens the generalizability of the results to the rest of the country.

Despite the methodological limitations of the study, we think it is likely that the results can be transferred to similar populations in other countries with similar societal, cultural and social conditions.

## Conclusions

The current study found that being bullied in early or late adolescence was related to depressive symptoms in adulthood, especially among those being bullied frequently and those who reported being bullied at both ages 15 and 18. This study adds to a growing body of research showing that being bullied in childhood can have potential serious mental health consequences in the longer run. Bullying should therefore be considered an important public health risk. Providing more detailed information to schools and local communities about the negative consequences of bullying and developing interventions to reduce bullying may help reduce the development of depressive symptoms later in life.

## Data Availability

The datasets used and/or analysed during the current study are available from the corresponding author on reasonable request.
